# Structural readiness to implement community-wide mass drug administration programs for soil-transmitted helminth elimination: results from a three-country hybrid study

**DOI:** 10.1186/s43058-021-00164-3

**Published:** 2021-07-19

**Authors:** Arianna Rubin Means, Elizabeth Orlan, Marie-Claire Gwayi-Chore, Angelin Titus, Saravanakumar Puthupalayam Kaliappan, Comlanvi Innocent Togbevi, Félicien Chabi, Katherine E. Halliday, Providence Nindi, Euripide Avokpaho, Khumbo Kalua, Moudachirou Ibikounlé, Sitara S. R. Ajjampur, Bryan J. Weiner, Judd L. Walson, Kumudha Aruldas

**Affiliations:** 1 Department of Global Health, University of Washington, Seattle, USA; 2 The Wellcome Trust Research Laboratory, Division of Gastrointestinal Sciences, Christian Medical College, Vellore, India; 3 Institut de Recherche Clinique du Benin, Abomey-Calavi, Benin; 4 Faculty of Infectious and Tropical Diseases, London School of Hygiene & Tropical Medicine, London, UK; 5 Blantyre Institute for Community Outreach (BICO), Lions Sight First Eye Hospital, Blantyre, Malawi; 6 College of Medicine, University of Malawi, Blantyre, Malawi; 7 Centre de Recherche pour la lutte contre les Maladies Infectieuses Tropicales (CReMIT/TIDRC), Université d’Abomey-Calavi, Abomey-Calavi, Benin

**Keywords:** Neglected tropical disease, Organizational readiness, ORIC, Organizational capacity

## Abstract

**Background:**

Current soil-transmitted helminth (STH) control programs target pre-school and school-age children with mass drug administration (MDA) of deworming medications, reducing morbidity without interrupting ongoing transmission. However, evidence suggests that STH elimination may be possible if MDA is delivered to all community members. Such a change to the STH standard-of-care would require substantial systems redesign. We measured baseline structural readiness to launch community-wide MDA for STH in Benin, India, and Malawi.

**Methods:**

After field piloting and adaptation, the structural readiness survey included two constructs: Organizational Readiness for Implementing Change and Organizational Capacity for Change. Sub-constructs of organizational readiness include change commitment and change efficacy. Sub-constructs of organizational capacity include flexibility, organizational structure, and demonstrated capacity. Survey items were also separately organized into seven implementation domains. Surveys were administered to policymakers, mid-level managers, and implementers in each country using a five-point Likert scale. Item, sub-construct, construct, and domain-level medians and interquartile ranges were calculated for each stakeholder level within each country.

**Results:**

Median organizational readiness for change scores were highest in Malawi (5.0 for all stakeholder groups). In India, scores were 5.0, 4.0, and 5.0 while in Benin, scores were 4.0, 3.0, and 4.0 for policymakers, mid-level managers, and implementers, respectively. Median change commitment was equal to or higher than median change efficacy across all countries and stakeholder groups.

Median organizational capacity for change was highest in India, with a median of 4.5 for policymakers and mid-level managers and 5.0 for implementers. In Malawi, the median capacity was 4.0 for policymakers and implementers, and 3.5 for mid-level managers. In Benin, the median capacity was 4.0 for policymakers and 3.0 for mid-level managers and implementers. Median sub-construct scores varied by stakeholder and country.

Across countries, items reflective of the implementation domain ‘policy environment’ were highest while items reflective of the ‘human resource’ domain were consistently lower.

**Conclusion:**

Across all countries, stakeholders valued community-wide MDA for STH but had less confidence in their collective ability to effectively implement it. Perceived capacity varied by stakeholder group, highlighting the importance of accounting for multi-level stakeholder perspectives when determining organizational preparedness to launch new public health initiatives.

**Trial registration:**

NCT03014167

Contributions to the literature

- This study describes the development of a structural readiness survey, including constructs for organizational readiness for change and capacity for change. These constructs are used to measure preparedness to deliver innovative community-based soil-transmitted helminths (STH) treatment programs.
- Organizational readiness and capacity for change vary by stakeholder group in this study, indicating that for system-wide interventions that require an array of stakeholders to effectively perform, it is necessary to consider multi-level preparedness for implementation.
- Study findings can guide change management activities targeted to specific stakeholders within a health system, increasing the likelihood of effective delivery of treatment campaigns with high coverage.

## Background

Soil-transmitted helminths (STH) are intestinal parasites that infect approximately 1.5 billion people globally, predominantly in low-and-middle-income countries (LMICs) [1]. Chronic and heavy STH infections are associated with diarrhea, malnutrition, and iron deficiency anemia, and other adverse outcomes including cognitive and growth delays in children [2]. In addition, STH perpetuate cycles of poverty by affecting children’s school attendance and performance and adult income-generating potential [3].

Because children and pregnant women experience the majority of STH-associated morbidity, current World Health Organization (WHO) guidelines recommend a control strategy predicated upon regular mass drug administration (MDA) of deworming medications delivered to pre-school and school-age children as well as other select high-risk groups such as women of reproductive age [4]. However, adults and untreated children continue to serve as reservoirs for the transmission of STH [5]. Recent studies demonstrate that community-wide MDA (cMDA) targeting individuals of all ages may be more effective than school-based MDA in reducing community-level STH infection prevalence and intensity and that such a strategy has the potential to interrupt STH transmission in some settings [6] A transmission interruption strategy for STH would require transitioning and restructuring from platforms that target and reach children through routine school-based MDA to platforms that reach entire communities through routine cMDA. This transition would involve substantial systems redesign, potentially including: transferring ownership of STH MDA programs from Ministries of Education to Ministries of Health, or establishing new coordination responsibilities across the two agencies; instituting new training mechanisms for volunteer community drug distributors (CDDs) to engage in cMDA; strengthening or forming new supply chains for the increased quantity of deworming medications needed to deliver cMDA; and adapting community sensitization resources and activities to reach community members of all ages, as opposed to only families with school-age children.

Organizational readiness assessments can measure the preparedness of the health system to engage in the launch of a new policy or program, such as cMDA for STH [7, 8]. Organizational readiness for change theory posits that readiness is reflected by the degree to which organizational members have a shared determination to implement change (i.e., change commitment) and a shared belief in their collective ability to implement change (i.e., change efficacy) [9]. These constructs are, in turn, influenced by the degree to which organizational members value the proposed change (i.e., change valence) and other informational assessments, such as the task demands and resources available to initiate the change [9]. Organizational assessments aim to determine if organizational members know what to do to implement a specific change, if members have the resources needed to enact the change, and if present circumstances are favorable for change (e.g., no major competing priorities) [9]. Organizational psychology suggests that implementation will be more effective when organizational readiness is high, as members will exert greater change-related effort, including engagement in change-related activities, dedication to change-related tasks, and cooperative behavior [9]. Assessing readiness and identifying barriers and facilitators is also in itself an implementation strategy, included in the Expert Recommendations for Implementing Change to improve uptake of evidence-based interventions [10].

We adapted an existing organizational readiness assessment tool and implemented surveys prior to the rollout of the DeWorm3 Project, a cluster-randomized control trial (cRCT) testing the feasibility of STH transmission interruption using cMDA in Benin, India, and Malawi [11]. We describe baseline preparedness to modify the current STH strategy and to launch a cMDA intervention for STH in each country. Because delivery of cMDA requires a cascade of activities from the national-level to the community-level, and a wide array of stakeholders to engage with specific responsibilities at each level, the “organizations” in this study consist of three separate stakeholder groups: policymakers, mid-level managers, and implementers. Evaluating readiness for this transition in strategy within each group could help identify organizational and health systems-wide determinants of preparedness and potential change management factors that might influence the adoption, implementation, and sustainability of new cMDA programs.

## Methods

This study is embedded within the DeWorm3 Project, a hybrid type I cRCT comparing primary outcomes of treatment uptake and STH infection prevalence between clusters randomized to cMDA or standard-of-care school-based MDA, with secondary assessments of factors influencing effective implementation [12]. DeWorm3 is described in detail elsewhere, including both clinical trial and implementation science protocols [1, 11]. Here, we evaluated readiness to implement cMDA prior to the rollout of the DeWorm3 project. We hypothesized that readiness would vary across countries and by stakeholder group within a country and, in particular, we expected to see the greatest variation between policymaker and implementer readiness.

### Survey development

We used the Organizational Readiness for Implementing Change (ORIC) tool as the basis of our assessment [13]. The ORIC is a 12-item survey developed and psychometrically validated in the United States (US) to determine the cognitive preparedness of organizational members to engage in a change in practice [13]. Compared to other organizational readiness measures, the ORIC was chosen for this study due to its demonstrated validity, reliability, and pragmatism. At the time of this study, the ORIC had only been used and validated in a US setting; therefore, prior to widespread use, we conducted a multi-step adaptation for use in LMIC settings.

Survey adaptations took place in Kenya in 2017 for three main reasons: STHs are endemic, Kenya has a similar neglected tropical disease (NTD) program design as Malawi and Benin, and a similar STH elimination study was ongoing there at the time, providing the opportunity to pilot survey items within a similar context for subsequent survey administration in Benin, India, and Malawi [6]. The first step of survey adaptation involved conducting cognitive interviews with key NTD stakeholders in Kenya (n = 3) to determine the interpretability and face validity of the ORIC questions. Minor adaptations were made to the wording of the ORIC survey based on these interviews and one item was removed from the survey due to poor interpretability (Appendix 1). Similar to the approach undertaken by prior ORIC development activities in the US, our adapted ORIC survey was then piloted in field-based simulations in Kwale, Kenya, with 140 local field researchers (all with a minimum secondary school education) involved in another STH elimination study [6, 13]. Field researchers were randomly assigned one of four vignettes, depicting a fictional health system with manipulated “high” or “low” levels of each organizational readiness construct: change commitment and change efficacy (Appendix 1). The field researchers completed an 11-item binary-response version of the ORIC survey after reading their assigned vignette. Qualitative reactions were also collected from the field researchers upon completing the surveys. Interviews were also conducted with global implementation partners (n = 5) involved in STH programming to identify any additional attributes of readiness that might influence effective delivery of cMDA for STH.

**Fig. 1:**
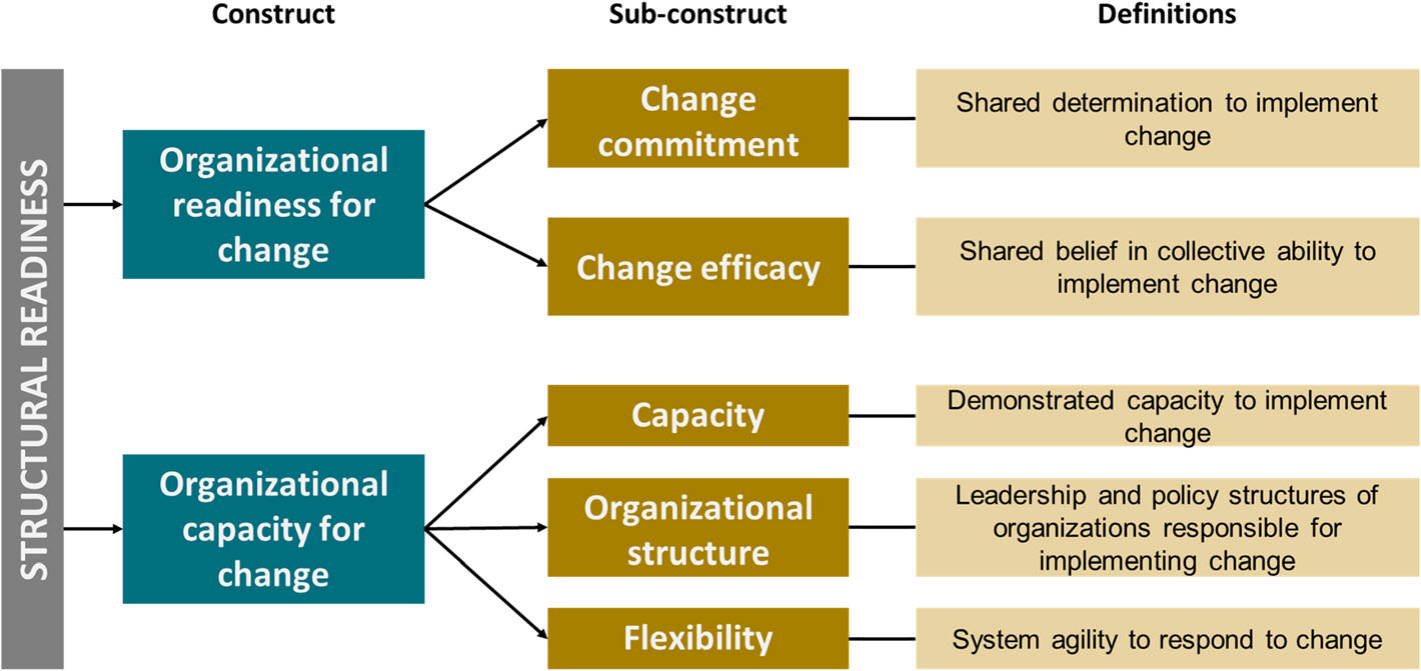
Structural readiness survey constructs, sub-constructs and definitions

The multi-step survey adaptation process demonstrated that, in addition to cognitive and behavioral drivers of readiness, the functional characteristics of health systems that influence the capacity to implement new innovations, such as supply chains and supervisory structures, are perceived to be equally important drivers of preparedness in LMICs. Based upon respondent feedback, the final survey included a module on the organizational capacity for change, with constructs including demonstrated capacity to implement, flexibility (a reflection of organizational agility to respond to changes), and organizational structure, including leadership and policy structures (Fig. 1). The multi-step adaptation also guided decisions to provide a five-point Likert scale response structured from highest to lowest preparedness: Strongly agree (5), Agree (4), Unsure (3), Disagree (2), and Strongly disagree (1). This decision to revert to using a Likert scale was driven by qualitative feedback from survey participants that they sought more nuance in readiness than a binary scale provided.

The adaptation process also informed the rewording of ORIC response options from the group-referenced “we” to “I”. For example, a change-commitment item reflecting motivation is worded as “I have observed that my co-workers are generally supportive of implementing cMDA for STH.” (Appendix 2). These adaptations reflected the discomfort described by health workers in reporting upon the readiness of their peers and supervisors, particularly within hierarchical health systems. The final adapted survey included 36 items, with 20 items measuring readiness for change and 16 items measuring capacity for change. Items were distributed according to sub-constructs of change commitment (n = 8), change efficacy (n = 12), demonstrated capacity (n = 6), flexibility (n = 4), and organizational structure (n = 6). Together, the readiness and capacity items comprise a newly envisioned structural readiness survey that accounts for both cognitive and infrastructural preparedness for change. An additional confirmatory item was included at the end of the survey that asked respondents to rate the overarching readiness of their organization to launch cMDA for STH.

We also categorized survey items into relevant health system implementation domains, apart from the readiness and capacity constructs in which they are originally assigned. These domains were conceptualized to provide more actionable evidence to stakeholders, including those at the participating countries’ Ministries of Health and Education, to guide programmatic decision-making. Domains included: policy environment (15 items), leadership structure (9 items), financial resources (7 items), material resources (3 items), human resources (3 items), technical capacity (3 items), and community delivery infrastructure (3 items).

Additional descriptive questions were added to the survey to determine participants’ institutional affiliation, level, role, familiarity with domestic STH policy and guidelines, experience working with CDDs, experience working in the areas of STH or other NTD research, as well as other indicators of STH and MDA program familiarity. The survey was forward-translated into French and Tamil for distribution in Benin and India, and backward-translated into English for quality assurance. Surveys were distributed in English to participants in Malawi.

### Sampling

Prior to this study, a stakeholder mapping exercise was conducted with policymakers and implementation partners in each country to identify all individuals who influence or would be influenced by a change in delivery of MDA for STH. Stakeholder mapping workshops included personnel from the Ministry of Health, Ministry of Education, and at least one representative from a community-based or non-governmental organization involved in the delivery of school-based or cMDA for STH. These maps provided the sampling frame for this analysis. Stakeholders were sampled from the maps based on three groupings: policymakers (WHO representatives, external implementation partners, and national and regional government officials who set national STH control guidelines and provide funding or technical support), mid-level managers (district level supervisors and managers who manage MDA planning and delivery), and implementers (healthcare workers, CDDs, and CDD supervisors who sensitize communities, deliver MDA and collect coverage data) within each country. We aimed to sample 10% of each stakeholder group identified in the stakeholder maps.

### Survey administration

A paper version of the final adapted survey was administered to sampled stakeholders prior to the launch of the cMDA program in each DeWorm3 site. Before administering the survey, trained survey administrators introduced the readiness survey using a standardized script to minimize systematic bias during administration.

Policymakers and mid-level managers were approached in their places of work and appointments were scheduled to administer the survey when participants were not immediately available. Participants in all sites had the option to self-administer the survey. Survey skip logic ensured that some capacity for change questions pertaining to national policy and administration were not asked of implementers, as implementers do not interface with these activities. . As a result, the survey ranged from 36 questions (policymakers and mid-level managers) to 24 questions (implementers).

### Analyses

Within each country, median scores and interquartile ranges (IQR) were calculated by stakeholder group for each item, sub-construct, construct, and implementation domain. Medians were used as opposed to means due to the small sample size in some stakeholder levels, and the presence of skewed (non-normally distributed) responses [14]. Respondents were required to answer all survey items; however, respondents were able to select ‘not enough information to answer’ for questions that they felt ill-equipped to answer. During analyses, responses of ‘not enough information to answer’ were combined with a Likert scale score of 3, corresponding qualitatively to “unsure,” as these responses reflect similar degrees of equanimity.

### Ethical approval

Survey participation did not require written or verbal informed consent. Before beginning, participants were informed—and checked a box indicating their agreement—that submission of their responses provided consent to participate. This study was reviewed and approved by the Institut de Recherche Clinique au Bénin (IRCB) through the National Ethics Committee for Health Research (002-2017/CNERS-MS) from the Ministry of Health in Benin, The London School of Hygiene and Tropical Medicine (12013), The College of Medicine Research Ethics Committee (P.04/17/2161) in Malawi, and the Institutional Review Board at Christian Medical College, Vellore (10392). The DeWorm3 Project was also approved by the University of Washington (STUDY00000180).

## Results

### Descriptive results

The adapted structural readiness survey was administered to 275 individuals, with 122 respondents in Benin, 74 in India, and 79 in Malawi, as detailed in Table 1. Participants were asked about their experiences with STH policy and programs, to contextualize structural readiness findings. Awareness of a national STH control policy was high among policymakers and mid-level managers. However, few of these stakeholders in Benin and India knew about the specific STH control policy in each country (14% and 25%, respectively), while knowledge of the country-specific policy was high in Malawi (100%).

**Table 1:** Demographic characteristics of survey respondents across Benin, India and Malawi (N=275)

Respondent characteristics	Benin (n=122)	India (n=74)	Malawi (n=79)
**Stakeholder level**			
Policymaker	**15**	**3**	**12**
*WHO*	2	1	
*National*	6	1	8
*Implementation partner*	2		4
*Regional*	5	1	
Mid-level manager	**9**	**5**	**10**
Implementer	**98**	**66**	**57**
*Health centers*	12	26	11
*CDD/ASHA/HSA*	86	40	46
**Knowledge of NTD/STH Master Plan**			
Believes country has a national policy for NTD control^a^	94.7%	75.0%	66.7%
Knowledge of NTD Master Plan^b^	95.5%	100.0%	80.0%
Identified correct STH policy outlined in Master Plan^c,d^	14.3%	25.0%	100.0%
**Familiarity with CDDs (%)**			
Worked as a CDD during previous MDA ^e^	83.7%	67.5%	89.1%
Experience training or supervising CDDs^f^	83.3%	79.0%^4^	51.5%
Works with CDDs^f^	86.1%	88.2%^4^	81.8%
**Perceived number of CDDs needed**^**g**^ **[mean (SD)]**			
Policymaker	17 (14.1)	8 (10.4)	18 (22.1)
Mid-level manager	59 (24.1)	26 (28.3)	15 (20.2)
Implementer	23 (24.3)	28 (22.0)	3 (1.7)
**Supervisory characteristics**			
Involvement in NTD research activities^f^	80.6%	79.4%	48.5%
Presents new ideas to supervisors^h^	55%	100%	94.4%
Oversees MDA activities at a local level	85.3%	77.0%	74.7%
**Material and resources needed for community-wide MDA (% of participants identifying)**^**)i**^			
Information Education Communication (IEC) materials	26.3%	25.2%	25.6%
Access to printing for field materials	15.6%	5.0%	3.0%
Incentives for CDDs	24.1%	21.6%	22.2%
Vehicles and fuel	1.1%	15.1%	11.1%
Well-trained staff	15.3%	21.1%	20.5%
MDA delivery guide for CDDs	12.9%	7.3%	6.8%
Other	1.4%	4.6%	6.0%
Missing	3.3%	0.5%	4.7%

^a^Asked to policymaker-level stakeholders (national, state, district, WHO, implementation partners)

^b^Among policymakers and mid-level managers who reported having knowledge of a national policy for the control of NTDs

^c^Of those who reported having knowledge of the NTD Master Plan

^d^The correct policy in Malawi is defined as annual national deworming of pre-school and school-age children at school, in India as bi-annual national deworming of pre-school and school-age children at schools and Angawadi centers, and in Benin as annual national deworming of enrolled children (ages 5–14 years)

^e^Measured only among CDDs

^f^Measured among all stakeholders except for CDDs

^g^Perceived number of CDDs needed to deliver MDA to a 5000-person catchment area

^h^Measured only among National and regional-level stakeholders

^I^Participants asked to identify three most critical material resources needed to deliver community-wide MDA

Across the three countries, most policymakers and mid-level managers had experience working with CDDs. In Benin (83%) and India (79%), policymakers and mid-level managers reported experience training or supervising CDDs; however, in Malawi, only 52% had experience working with CDDs in this capacity. In each country, over three-quarters of stakeholders reported supervising or engaging in MDA activities at a local level, indicating a familiarity with the realities of implementing MDA. Similarly, most of the CDDs in each country had previous experience supporting MDA delivery in Malawi (89%), Benin (84%), and India (68%).

Stakeholders were asked about their perceptions regarding the inputs required to deliver cMDA with high coverage, including the number of CDDs and types of material resources needed to deliver MDA. There was a great deal of between-country and between-stakeholder variation in the perceived number of CDDs required to deliver MDA for a catchment area population of 5000 people, ranging from implementers in Malawi (average: 3) to mid-level managers in Benin (average: 59). When asked what materials were most important for delivering cMDA, participants identified community education materials as most important (25–26%), CDD incentives (22–24%), and well-trained staff (15–21%).

### Benin results

**Fig. 2:**
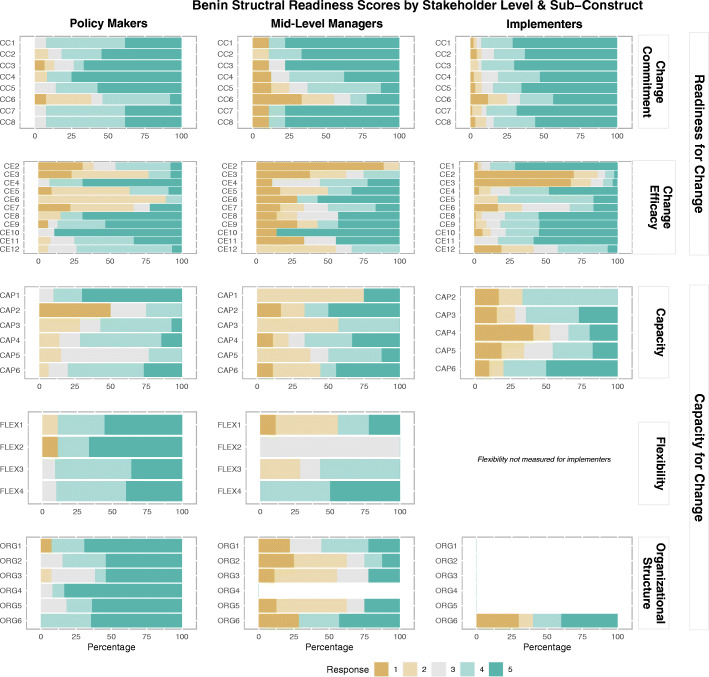
Benin structural readiness responses by sub-construct and stakeholder level (note: item-level acronyms can be found in Appendix 4 in Table 11)

Overall median readiness for change was 4.0 for both policymakers and implementers and 3.0 for mid-level managers, indicating that policy makers and implementers “somewhat agreed” that Benin is ready to launch cMDA, while mid-level managers were “unsure”. Median change commitment in Benin was lowest among policymakers, compared to other stakeholders (Table 2, Fig. 2). Median change efficacy scores were lowest for mid-level managers (4.0).

**Table 2:** Median readiness scores by structural readiness constructs and sub-constructs (N=275)

Construct	Subconstruct	Benin (median and IQR) (n=122)			India (median and IQR) (n=74)			Malawi (median and IQR) (n=79)		
		Policymakers (n=15)	Mid-level managers (n=9)	Implementers (n=98)	Policymakers (n=3)	Mid-level managers (n=5)	Implementers (n=66)	Policymakers (n=12)	Mid-level managers (n=10)	Implementers (n=57)
**Organizational readiness**		**4.0 (1.0)**	**3.0 (1.0)**	**4.0 (1.0)**	**5.0 (0.5)**	**4.0 (1.0)**	**5.0 (0.5)**	**5.0 (1.0)**	**5.0 (1.0)**	**5.0 (1.0)**
	**Change commitment**	**4.0 (2.0)**	**4.5 (1.0)**	**5.0 (1.0)**	**5.0 (0)**	**5.0 (0)**	**5.0 (0)**	**5.0 (0)**	**5.0 (0)**	**5.0 (0)**
	Needed	4.0 (1.0)	5.0 (0.5)	5.0 (1.0)	5.0 (0)	5.0 (0.5)	5.0 (0)	5.0 (0)	5.0 (0)	5.0 (0)
	Motivated	4.5 (1.5)	3.5 (2.0)	4.5 (1.0)	5.0 (0)	5.0 (0)	5.0 (0)	5.0 (0.3)	5.0 (0)	5.0 (0.5)
	Outcome expectancies	4.0 (2.0)	4.5 (1.0)	4.5 (1.5)	5.0 (0)	5.0 (0.5)	5.0 (0.5)	4.8 (0.5)	5.0 (0)	5.0 (0)
	**Change efficacy**	**4.0 (1.0)**	**3.0 (1.0)**	**4.0 (1.0)**	**4.0 (2.5)**	**4.0 (0)**	**4.5 (1.5)**	**4.0 (0)**	**3.0 (1.0)**	**3.5 (1.0)**
	Task demand	4.0 (2.0)	2.0 (2.0)	1.0 (2.0)	4.0 (1.0)	2.0 (1.0)	3.0 (2.0)	4.0 (3.5)	3.0 (3.0)	3.0 (1.5)
	Resource availability	3.5 (1.5)	3.0 (1.0)	4.0 (1.5)	4.0 (3.0)	4.0 (0)	4.5 (1.5)	3.5 (1.0)	3.5 (1.0)	3.0 (1.5)
	Contextual factors	4.0 (0.5)	3.0 (3.0)	3.8 (1.0)	4.5 (1.0)	4.5 (1.0)	4.5 (1.0)	4.0 (0.8)	4.0 (0.5)	4.0 (1.5)
**Organizational capacity for change**		**4.0 (1.0)**	**3.0 (1.0)**	**3.0 (2.0)**	**4.5 (1.0)**	**4.5 (1.0)**	**5.0 (1.5)**	**4.0 (0.5)**	**3.5 (1.0)**	**4.0 (1.5)**
	**Demonstrated capacity**	**3.5 (1.0)**	**3.0 (2.0)**	**3.0 (2.0)**	**3.0 (1.0)**	**4.5 (1.0)**	**5.0 (2.0)**	**3.5 (0.8)**	**3.8 (1.0)**	**4.0 (1.0)**
	**Flexibility**	**4.0 (2.0)**	**3.5 (0.5)**		**5.0 (0)**	**4.5 (0.5)**		**3.5 (1.5)**	**3.3 (1.5)**	
	**Organizational structure**	**5.0 (1.0)**	**2.0 (2.0)**	**3.5 (3.5)**	**4.5 (1.5)**	**5.0 (2.0)**	**5.0 (0)**	**4.5 (1.8)**	**4.5 (2.0)**	**5.0 (1.0)**
	Leadership structure	5.0 (1.0)	2.0 (2.0)		4.0 (1.0)	5.0 (2.0)		5.0 (0.5)	4.5 (2.0)	
	Political structure	5.0 (1.0)	3.5 (1.0)	3.5 (3.5)	5.0 (2.0)	4.0 (1.0)	5.0 (0)	5.0 (1.8)	4.3 (2.5)	5.0 (2.0)

Bold: combined median scores of subconstructs below

The overall median organizational capacity for change score was highest for policymakers (4.0), and lower for mid-level managers and implementers (3.0), indicating that policymakers “somewhat agree” that Benin has the capacity to launch cMDA while mid-level managers and implementers are “unsure”. Median scores for demonstrated capacity were slightly higher for policymakers as compared to other stakeholders. For the construct of flexibility, median scores were also higher among policymakers (note that this sub-construct was not measurable for implementers, as described above). Median scores for the organizational structure were lowest for mid-level managers, as compared to other stakeholders. The difference in organizational structure scores is largely reflective of differing responses to items about the effectiveness of program leadership at regional and district levels.

When asked to provide an overarching assessment of preparedness to implement cMDA for STH, implementers reported the highest preparedness (5.0) compared to policymakers (4.0) and mid-level managers (3.0) (Appendix 3 in Table 8). With the exception of mid-level managers, who reported that they were “unsure” if Benin was ready to implement cMDA, these scores are more optimistic (higher) than stakeholder-specific summary scores at the construct levels.

Median readiness scores were also calculated for specific implementation domains to identify strengths and weaknesses in the health system’s preparedness to deliver cMDA (Table 3). Observed readiness in the domains of policy environment and technical capacity were high (4.0 or above) across stakeholders. For health system leadership, mid-level managers reported the lowest scores. Material resources were also scored lowest by mid-level managers (3.0) Policymakers and mid-level managers scored the domain of financial resources lower than implementers. Human resources were scored highest among policymakers (3.0) and extremely low by implementers (1.0), who are the primary implementation workforce for cMDA. The domain of community delivery infrastructure was also highest among policymakers who are, arguably, most removed from this infrastructure on a day-to-day basis.

**Table 3:** Median readiness scores by health system implementation domains

Domain	Benin (median and IQR)			India (median and IQR)			Malawi (median and IQR)		
	Policymakers	Mid-level managers	Implementers	Policymakers	Mid-level managers	Implementers	Policymakers	Mid-level managers	Implementers
**Policy environment**	4.0 (2.0)	4.5 (2.0)	4.5 (1.0)	5.0 (0)	5.0 (0)	5.0 (0)	5.0 (0)	5.0 (0)	5.0 (0)
**System leadership**	4.0 (1.0)	3.0 (0.5)	4.5 (1.0)	5.0 (1.0)	5.0 (1.0)	5.0 (0.5)	5.0 (1.0)	4.3 (1.5)	5.0 (0.5)
**Financial resources**	3.0 (1.0)	3.0 (2.0)	3.5 (1.0)	3.0 (2.0)	4.0 (0)	3.5 (1.5)	3.0 (1.0)	3.0 (1.0)	2.0 (1.0)
**Material resources**	4.0 (1.0)	3.0 (1.0)	4.0 (1.5)	2.0 (3.0)	5.0 (0)	4.5 (1.5)	4.0 (1.0)	3.5 (3.0)	3.5 (1.5)
**Human resources**	3.0 (2.0)	2.0 (2.0)	1.0 (1.0)	1.0 (3.0)	2.0 (0)	1.5 (1.0)	2.5 (3.0)	2.5 (3.0)	1.0 (1.0)
**Technical capacity**	4.0 (1.0)	4.0 (1.0)	4.0 (2.0)	4.0 (2.0)	4.0 (1.0)	5.0 (1.0)	3.5 (1.3)	4.0 (1.0)	3.5 (1.5)
**Community-delivery infrastructure**	4.0 (2.0)	3.0 (3.0)	3.5 (1.0)	4.0 (2.0)	5.0 (1.0)	5.0 (1.0)	5.0 (0)	5.0 (1.0)	3.5 (1.5)

### India results

**Fig. 3:**
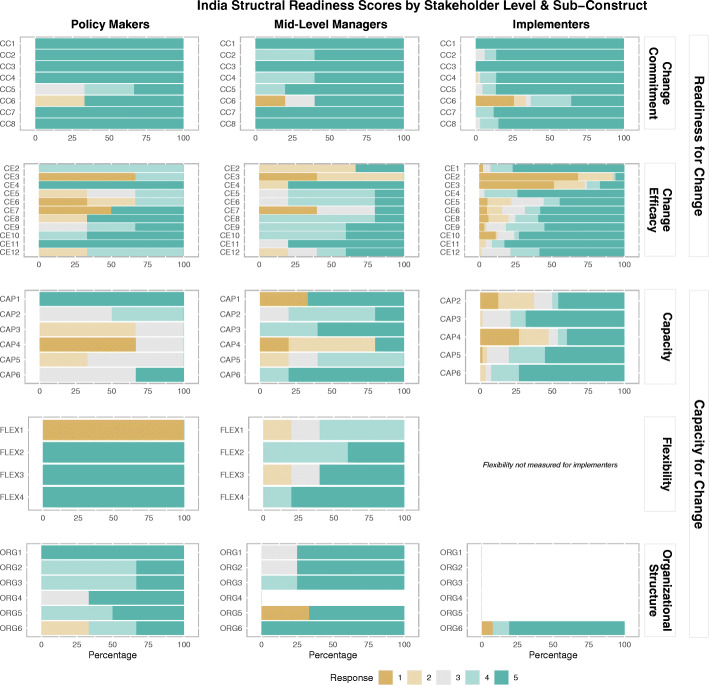
India structural readiness responses by sub-construct and stakeholder level (note: item-level acronyms can be found in Appendix 4 in Table 11)

Overall median organizational readiness for change in India was highest for policymakers and implementers (5.0) and lower for mid-level managers (4.0), indicating that policymakers and implementers “agreed” that India is ready to launch cMDA and mid-level managers “somewhat agreed” (Table 2, Fig. 3). All stakeholder groups reported high median change commitment (5.0). Change efficacy was lower for policymakers and mid-level managers (4.0) as compared to implementers (4.5).

The overall organizational capacity for change median scores were 4.5 for both policymakers and mid-level managers and 5.0 for implementers, indicating that stakeholders generally agreed that India has the capacity to launch cMDA. Median demonstrated capacity scores were lower for policymakers. Median organizational structured scores were also lowest among policymakers. However, median flexibility scores were highest for policymakers indicating that policymakers perceive flexibility in the existing MDA infrastructure.

When asked to provide an overarching assessment of preparedness to implement cMDA for STH, policymakers indicated lowest preparedness (3.0) compared to the mid-level managers and implementers (5.0) (Appendix 3 in Table 9). This score reflects less optimism among policymakers in India, than what is observed in their readiness for change and capacity for change scores alone.

When survey results were organized by implementation domain, all stakeholders identified high readiness within the policy environment (Table 3). Median scores for the domain of technical capacity were highest among implementers, compared to other stakeholders, while all stakeholders perceived health system leadership to be high. Readiness driven by financial resources was lowest for policymakers compared to other stakeholders. Perceived readiness driven by human resources was low among all stakeholders, but lowest for policymakers. Likewise, median readiness driven by material resources and community delivery infrastructure was also lowest among policymakers.

### Malawi results

**Fig. 4:**
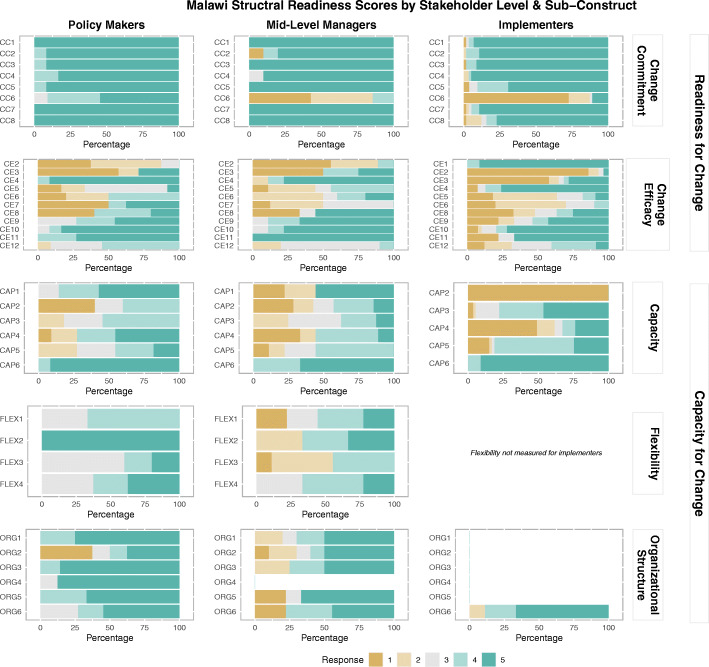
Malawi structural readiness responses by sub-construct and stakeholder level (note: item-level acronyms can be found in Appendix 4 in Table 11)

Overall organizational readiness for change was high in Malawi, with a median score of 5.0 for all stakeholder groups (Table 2, Fig. 4), indicating agreement that Malawi is ready to launch cMDA for STH. Median change commitment was high across stakeholders at 5.0. Median change efficacy was lowest for mid-level managers (3.0).

The overall organizational capacity for change median scores were 4.0 for policymakers and implementers, and 3.5 for mid-level managers, indicating that most stakeholders “somewhat agree” that there is the capacity to launch cMDA for STH in Malawi. Like India, median scores for the sub-construct of demonstrated capacity were lower for policymakers as compared to other stakeholders. Median flexibility sub-construct scores were similar (around 3.5) for all stakeholders. Median organizational structure sub-construct scores were highest for implementers in Malawi.

Across stakeholder groups, when asked to provide an overarching assessment of preparedness to implement cMDA for STH, responses were consistently high (5.0) for all groups (Appendix 3 in Table 10). This high readiness mirrors overall organizational readiness scores and is more optimistic than overall organizational capacity scores.

By implementation domain, readiness depicted by the policy environment domain was high across all groups (5.0) (Table 3). Within the technical capacity domain, mid-level managers had slightly higher median readiness scores compared to other stakeholders. Similar to findings from Benin, mid-level managers in Malawi reported the lowest median scores for the leadership domain. Median readiness scores were lower for all stakeholders for the domain of financial resources as compared to other implementation domains (3.0 for policymakers and mid-level managers; 2.0 for implementers). Median readiness scores within the human resources domain were similarly low for all stakeholders (2.5 for policymakers and mid-level managers, 1.0 for implementers). Median readiness within the material resources domain and the community-delivery infrastructure domain were highest for policymakers as compared to other stakeholders.

### Summary of findings

Across all sites, capacity for change was lower than readiness for change for all stakeholders (except mid-level managers in India), indicating that stakeholders may be cognitively prepared to launch cMDA for STH, but believe they lack the structural resources and infrastructure needed to do so. Similarly, change efficacy, a sub-construct of organizational readiness for change, was lower than change commitment, indicating some doubt in the respondent’s collective ability to effectively implement cMDA. Across countries, many constructs, including change efficacy, were lower for mid-level managers, driving lower organizational readiness and capacity for change scores among these stakeholders, specifically.

## Discussion

This study describes and compares preparedness to launch cMDA for STH in three LMICs prior to their engagement in targeted cMDA activities, using a novel structural readiness survey that combines measures of organizational readiness with measures of organizational capacity for implementing change. Overall, structural readiness to launch cMDA for STH was high across the surveyed stakeholders in Benin, India, and Malawi. However, within each country and stakeholder group, there were notable variations in structural readiness by construct, sub-construct, and implementation domains. These observed variations in preparedness are meaningful within a context of otherwise generally high readiness to transition to a cMDA strategy for STH elimination, and eventual intervention scale-up.

### Organizational readiness for change

In all countries, median change commitment, a sub-construct of organizational readiness for change, was consistently high across stakeholder groups while change efficacy was slightly lower and varied by stakeholder. Other studies from high-income countries have similarly found change commitment to be higher than change efficacy during pre-intervention readiness assessments [15–17]. As conceptualized in the creation and validation of the ORIC, change commitment is expected to be high in contexts where organizational members have a shared interest in making a change. Change efficacy is expected to be high when organizational members know what to do and how, when they have the necessary resources to change, and when environmental factors are also amenable to the change [13]. Findings from our study indicate that there is a perceived need and interest in implementing cMDA for STH transmission interruption, but lower confidence in collective ability to effectively implement cMDA for STH transmission interruption.

In this study, lower change efficacy scores were driven by responses to items regarding the need for more training, more human resources to support coordination and supervision, and expectations of resource availability. In Benin and Malawi, change efficacy scores were lower among mid-level managers compared to other stakeholders. These lower scores were driven by responses to questions about training, supervision, perceived community resistance, and challenges moving financial resources across administrative levels. The lower change efficacy among mid-level managers in Benin and Malawi compared to other stakeholder levels highlights the unique role of these stakeholders in program implementation. The personnel link between policymakers and implementers, mid-level managers may be particularly aware of multi-level challenges to program delivery.

Similarly, in India, both mid-level managers and implementers had particularly low responses to questions about training and supervisor coordination. These concerns may influence change efficacy by reducing stakeholders’ confidence that MDA can be delivered with high treatment coverage to targeted populations. A systematic review of lymphatic filariasis (LF) cMDA programs found that inadequate CDD training might negatively affect community member participation in MDA programs by compromising CDD communication skills, credibility, and knowledge about the drugs being distributed [18]. Likewise, CDD motivation and, in effect, MDA treatment coverage is positively affected by supervision and support from NTD program supervisors [19]. Across the countries, investing in CDD supervisory structures and training may similarly have a positive impact on MDA treatment coverage by influencing the perceived preparedness of stakeholders closest to implementation.

### Capacity for change

We defined capacity for change as a collection of demonstrated organizational capacity, organizational flexibility, and organizational structure. Given that there was not an existing validated scale for organizational capacity in an LMIC at the time of the study, we created sub-scales for each construct based on feedback provided during adaptation activities conducted in Kenya. In contrast to organizational readiness constructs, capacity for change constructs exhibited more variability by stakeholder and country, with the lowest capacity scores for mid-level managers in Benin and the highest for policymakers and mid-level managers in India. Lower capacity for change scores in Benin were driven by responses to items about observed delays in receipt of MDA medicines and observed challenges implementing NTD programs, including the NTD Master Plan, in country. The observed variability in the capacity for change sub-constructs across countries and stakeholders may be due to the fact that the items could be answered objectively based on each respondent’s experiences and observations of their organization and treatment programs generally, with less personal self-reflection and judgment of their colleagues as compared to the organizational readiness for change constructs.

In Malawi, the capacity for change sub-construct of system flexibility, or agility to adapt when delivering new interventions, was particularly low. Notably, mid-level managers reported that their supervisors did not feel comfortable receiving feedback, and policymakers reported similar sentiments. Nearly 95% of participating policymakers and mid-level managers in Malawi reported that they have tried to present new ideas to their supervisors in the past. The ability to provide feedback and shape the course of an organization is fundamental to organizational identity and may drive, in effect, stakeholder motivation.

Mid-level managers in Benin scored items in the sub-construct of organizational structure lower than other stakeholders. In particular, mid-level managers expressed doubt that MDA programs are effectively implemented at the regional and district levels. Mid-level managers also indicated that the NTD Master Plan does not provide sufficient guidance for delivering cMDA programs (such as MDA for LF). This suggests the need for change management activities that orient all personnel, including mid-level managers and implementers, to the disease-specific policy and guidelines. This change management activity could provide the opportunity to enhance team cohesiveness and preparedness prior to future intervention implementation. Low-cost change management activities, such as building strategy and priority consensus across health workers, are particularly important in NTD-endemic countries where resources are often limited.

### Implementation domains

We also summarized and presented the data according to the implementation domains associated with each survey item. In doing so, we aimed to share these data in an easily interpretable format with Ministries of Health and other collaborators who need to rapidly assess preparedness to roll out a change in practice. In general, domains related to the policy environment and technical capacity were rated highest across stakeholders and countries when compared to other domains. Variations in perceived preparedness across other domains may reflect the different roles, responsibilities, and insights associated with each stakeholder’s role. For example, in Malawi, items reflective of “community delivery infrastructure” were rated higher among policymakers and mid-level managers and lower among implementers, who are closest to delivery on the ground. Across all countries and stakeholders, items reflective of the “human resources” domain were scored low, representing a key potential weakness in NTD programs that largely rely upon a volunteer workforce. Items reflective of “financial resources” were also lower than other domains, for most stakeholders and countries. Descriptive data from the survey indicate that participants were uncertain about what financial incentives CDDs receive, with a wide range of incentives reported by implementers themselves (e.g., in Malawi estimates ranged from $4.07–$33.95 USD per day). This lack of agreement on incentivization amount, but consensus regarding incentivization inadequacy, may reflect a generality that implementers feel undervalued. Both financial and non-financial incentives (e.g., refreshments at trainings or training certificates) may contribute to increasing the perceived value associated with CDD responsibilities and, subsequently, increased preparedness. Organizing health system readiness data by implementation domain may be a useful way of communicating data about readiness and capacity to non-academic audiences, as the domains provide an alternate way of combining cognitive and infrastructural drivers of system preparedness. Yet, important nuance is missed at the domain level as compared to the constructs of readiness and capacity for change. This nuance may be necessary for identifying specific areas where preparedness to launch a new intervention is particularly strong or particularly tenuous.

### Change management

Change management approaches, such as improved training, supervision, and financial management can be used to address and improve organizational readiness to launch a new program or intervention [10]. Change management activities needed to optimize the rollout or scale-up of an innovation appear to necessarily vary by country and stakeholder level. For example, in India, policymakers were less confident than implementers regarding items related to recruitment of CDDs and delays in drug arrival. In comparison, in Malawi, implementers were less confident than policymakers about recruitment of CDDs. This is likely due to varying levels of experience with planning and implementation among the stakeholder groups, as well as differing responsibilities among the stakeholder groups, by country. Because there is contextual variation by stakeholder-level, tailoring change management or selecting implementations strategies tailored to the needs of stakeholder groups could help to optimize timing and deployment of resources prior to implementation of a novel program or innovation [20].

Other studies have incorporated assessments of capacity within a paradigm of readiness to change [21]. The R = MC^2^ heuristic defines organizational readiness as a product of an organization’s motivation to implement an innovation, in addition to their general capacity and innovation-specific capacity. Studies that have applied this framework in the USA found that addressing barriers to readiness may lead to improvements in both motivation and capacity to effectively implement the intervention [22]. In LMICs, where readiness and commitment to implement an innovation may not equate with capacity or resources to do so, it may be particularly important to add capacity measures to readiness assessments to fully understand organizational preparedness.

### Limitations

This study has several limitations. While the structural readiness survey was administered prior to the launch of the DeWorm3 Project, participants were aware of the imminent rollout of the intervention. As a result, social desirability bias may have influenced responses. Only three policymakers from India were included in this analysis and, given the large size of the Indian national government, these results are not generalizable to all policymakers. Also, because the survey was fairly long survey fatigue could have impacted responses. Additionally, we did not collect identifiable demographic information from participants such as age or sex, precluding disaggregation of data by these demographic characteristics. However, it was important to ensure participant anonymity given the sensitivity of some of the questions.

Additionally, during cognitive interviews and pilot tests of the ORIC items, we found that the use of the word “we” was uncomfortable for the respondents, as they did not want to speak for their colleagues. Thus, we used “I,” or “in my experience” statements. Other recent studies that have adapted the ORIC for use in other language and country contexts have also adjusted question phrasing to fit the needs of the population surveyed [23]. However, it is possible that these adjustments to the language inherently alter the meaning and value processing of survey respondents. Similarly, it is possible that observed differences in readiness across countries are linked to culturally-based differences in the interpretation of survey questions. A full assessment of the reliability and validity of the capacity for change items was not conducted during this survey administration because, pragmatically, the survey needed to be implemented prior to the rollout of a new intervention which did not allow for a lengthy psychometric validation. An additional assessment of structural readiness to scale-up cMDA will be conducted at the endline of DeWorm3, providing the opportunity to evaluate the reliability and validity of structural readiness constructs in an LMIC. Findings from this assessment may help determine if the survey can be applied to other interventions, outside of cMDA for STH.

## Conclusion

Overall organizational readiness to implement changes to current STH programs was high for most stakeholders across Benin, India, and Malawi and the construct of change commitment was higher than change efficacy across all countries. Organizational capacity for change was generally highest among policymakers across all countries, compared to mid-level managers and implementers, with heterogeneity in readiness sub-constructs across stakeholders. These analyses also identified more impenetrable barriers to structural readiness to deliver cMDA, including obstacles related to human, financial, and material resources. Resource scarcity is a common challenge across LMICs but is particularly notable within an STH program which typically benefits from a largely volunteer CDD workforce to distribute drugs in the community and drug donation programs that provide medicines to endemic countries at no cost. While these barriers can be addressed to some degree using efficiency planning to avoid activity duplication or through incentivization schemes that attract and maintain a strong CDD workforce, resource challenges are typically more systemic. Government policymakers and researchers may need to work together to understand if and how programs can implement cMDA for STH when organizational readiness, capacity to implement, or both are comprised.

This study is the first, to our knowledge, to apply an adapted version of the ORIC in an LMIC public health setting. It is also one of the first to evaluate the item and construct-level responses by multi-level stakeholder groups. For complex interventions that involve a cascade of activities and high performance of stakeholders across a health system, these findings suggest that it is important to understand how readiness and capacity to implement differs across stakeholders, rather than pooling responses or assuming that one group may be representative of all groups involved. When time and resources allow, readiness assessments can be used to guide diagnostic appraisals of programmatic strengths and weaknesses about the organizations’ preparedness to launch an intervention. For STH programs, a pre-intervention readiness assessment may be used to understand structural barriers to effective launch or scale-up of MDA with high treatment coverage, increasing the likelihood that cMDA delivery can, as a result, lead to successful disease elimination.

## Data Availability

The datasets used and/or analyzed during the current study are available from the corresponding author on reasonable request.

## Appendix

### Appendix 1: Overview of ORIC adaptation process

The survey adaptation process for this study was conducted in two parts. First, face validity assessments using cognitive interviews were conducted in order to evaluate the comprehension of survey questions [12]. Second, a field-based simulation was conducted to assess construct validity at the survey item and organizational levels.

#### Cognitive Interviews

Participants included a convenience sample of three Kenyan stakeholders: two national stakeholders within the Ministry of Health (MOH) and one county-level stakeholder from a non-governmental organization providing technical implementation support for the ongoing National School-Based Deworming Programme. Interviews took place over phone and Skype, lasting approximately one hour. Oral consent was received prior to initiating the interview. Participants were asked to respond to the survey within the context of implementing a future community-wide STH MDA campaign in-country. The ORIC survey included 10 binary response items (agree/disagree) measuring four change commitment items, six change efficacy items, and one item measuring overall readiness. A cognitive assessment interview guide was used to probe interview participants to describe their thought processes, interpretation, and ultimate responses for each survey item (Appendix 1 in Table 4). Detailed notes were taken to inform survey revisions (Appendix 1 in Table 5).

#### Psychometric Validation

Informed by a prior ORIC validation, four vignettes were developed depicting a fictitious country’s health system readiness to deliver community-wide MDA for STH. Vignettes are a commonly used and effective method in psychological research to assess validity survey item validity [13, 14]. They are considered more realistic and less abstract evaluation approaches than conventional survey methodologies and also allow for the simultaneous examination of the varied constructs embedded in the vignette scenarios [13]. A scenario was presented in each vignette where health system readiness was manipulated by altering specific indications of change commitment and change efficacy as “high,” “low,” or “moderate” (Appendix Tables 1C). For example, in Vignette 2, human resource challenges reflected low change efficacy while intense interest in community-wide MDA by MOH leadership reflected high change commitment.

Study participants included a convenience sample of 140 field workers and data officers based in Kwale County, Kenya employed by a randomized trial evaluating the impact and cost-effectiveness of alternative deworming delivery strategies in reducing STH transmission [15]. All field workers had completed a minimum of secondary school, meeting educational qualifications necessary to engage as validation study participants [10].

Each participant was randomly assigned to receive one vignette and then complete the ORIC survey independently as they believed that personnel within the fictional health system would respond. Responses were binary (agree/disagree). Participants were asked to project themselves into the vignette scenarios and not draw from their background or current work to inform their responses. Two versions of the survey were distributed with differently ordered questions to mitigate potential response biases. Written consent to participate was provided and all responses were anonymous.

Statistical analysis was conducted using STATA 13.1 [16]. A mean score for each ORIC item was calculated for each vignette to assess validity at the item level. Organizational-level validity was assessed by evaluating how scores performed across an entire vignette. Thresholds for mean readiness levels were defined *a priori* as: “low” readiness (1.00–1.35), “moderate/unknown” readiness (1.36–1.65), and “high” readiness (1.66–2.00). The percentage of items whose score fell within the expected thresholds of “low” or “high” readiness was calculated. Following survey administration, field workers participated in an informal focus group discussion to assess the survey administration process.

**Table 4:** Cognitive Interview Guide Questions

Assessment Indicator	Question During Interview
Comprehension	What is your initial response to the statement?
Intent	What do you think the statement appears to be referring to?
Meaning of terms	What do *specific word/phrase* in the statement mean to you?
Comprehension	Are there any words that are confusing or unclear?
Decision process	Is this statement easy or hard to respond to? What made it easy/hard?
Recallability of information and recall Strategy	What types of knowledge or information do you feel you need to pull from in order to respond to the statement?

**Table 5:** Modifications to ORIC Survey from Cognitive Interviews

Original item	Modified item	Rationale for modification
“We can *manage the politics* of implementing the transition from school-based MDA to community-wide MDA for STH”	We can *manage the coordination of all stakeholders involved* in implementing the transition from school-based MDA to community-wide MDA for STH.	Word use of “politics” perceived to be related to governance instead of internal management as expected. Perception may create undue tension in typically politically sensitive settings as implementation occurs within government structures. Respondent 1: “The word politics stands out as a risky word especially if administering the survey to government officials. This is setting yourself for politics-centered discussions because there may be things that are not resolved i.e. internal wars. [This item] is setting the respondents up to become engaged in politics.” Respondent 2: “Politics is a word that should be avoided. Try to focus on the setting or health climate. This can be tricky especially if there are elections happening. Some people will not respond to this because they fear that they will be talking about their bosses.”
We can *handle the challenges* that might arise in implementing community-wide MDA for STH.	We can *manage the challenges* that might arise in implementing community-wide MDA for STH.	The wording of “handle challenges” did not resonate contextually with respondents. Respondent 1: The word ‘handle’ is difficult. Change it to ‘manage’ as it…links to coordination and collaboration. If you say handle, then saying it is sole responsibility [of one person] but if you say manage, can bring in someone else to help overcome [issues].”
We can *keep momentum going* while implementing community-wide MDA for STH	Remove	The wording of “keep momentum going” did not resonate contextually with respondents and was too confusing and vague. Respondent 1: Momentum of what? When we say keep momentum going – where is responsibility being set? We can sustain periodic delays to implementation as long as it is required in country.” Respondent 3: What is momentum? Do you mean while implementing? What are we talking about? Can mean so many things [such as] momentum with integrating other programmes and partners or managing aspects of same programme.”

### Appendix 1C Vignettes used for field piloting

*Vignette 1: High change commitment, high change efficacy (high overall readiness)*

**Table:**

In Bahari, a fictional country in East Africa, many people are infected with intestinal worms. As recommended by the World Health Organization, the country’s focus has been on administering deworming medications to children in primary schools. However, as a result, many adolescents and adults have not been dewormed. The number of worm infections in Bahari is not decreasing and government officials believe it may be due to ongoing infections in these older age groups. The Ministry of Health (MOH) has decided that it will launch a new community-wide deworming campaign to make sure that everyone in the population—children, adolescents, and adults—is dewormed. The government’s goal is to stop the spread of intestinal worm infections and to improve the overall health of the community.	
Jane Daktari, the director of the MOH in Bahari, expressed confidence that the community-wide campaign will be a success. She said, “I am very keen about this new campaign because we know that providing deworming to all of the members in our community will be an effective way to improve health outcomes.” Mary Kitenge, a County-level MOH officer, agreed. She said, “I have talked with many local health system personnel and community leaders and there is widespread interest in launching a community-wide deworming campaign. Everyone is on board.” John Kanga, the Neglected Tropical Disease Programme Manager in Bahari said, “We have been implementing the school deworming campaign for several years now and will use that experience to make sure the community-wide campaign is a success. We have the necessary resources, as the national MOH has allocated funding in its annual budget to include the community-wide deworming campaign and we have a large number of community health workers who can administer the deworming medicines.” “This is a high priority campaign for the Ministry so we will be sure to carefully schedule the campaign until after the rainy season for ease of implementation,” said Daktari.	

*Vignette 2: High change commitment, low change efficacy (moderate overall readiness)*

**Table:**

In Bahari, a fictional country in East Africa, many people are infected with intestinal worms. As recommended by the World Health Organization, the country’s focus has been on administering deworming medications to children in primary schools. However, as a result, many adolescents and adults have not been dewormed. The number of worm infections in Bahari is not decreasing and government officials believe it may be due to ongoing infections in these older age groups. The Ministry of Health (MOH) has decided that it will launch a new community-wide deworming campaign to make sure that everyone in the population - children, adolescents, and adults – is dewormed. The government’s goal is to stop the spread of intestinal worm infections and to improve the overall health of the community.	
Jane Daktari, the director of MOH in Bahari, expressed confidence that the community-wide campaign will be a success. She said, “I am very keen about this new campaign because we know that providing deworming to all of the members in our community will be an effective way to improve health outcomes.” Mary Kitenge, a county-level MOH officer, agreed, saying, “I have talked with many local health system personnel and community leaders and there is widespread interest in launching a community-wide deworming campaign. Everyone is on board.” John Kanga, the Neglected Tropical Disease Programme Manager in Bahari said, “We have been relying on technical partners to implement the school deworming campaign for several years now. We at MOH will have to learn how to do the community-wide campaign on our own so that we can make sure it is a success. That could be challenging” Mr. Kanga will be transferred to a different department within the Ministry soon and there will be a new NTD manager who needs to manage the campaign. “The first thing this person will need to do is ensure that we have all of the necessary resources to make sure that everyone is reached. The national MOH has not allocated funding in its annual budget to include the community-wide deworming campaign. Also, I am concerned that we do not have enough deworming medicines in the country for such a large-scale campaign,” said Mr. Kanga. Ms. Kitenge said, “Interest in community-wide deworming is high, but we do not have enough existing community health workers who are ready to begin administration. Even if we hire enough, we will first need to focus on the immunization campaign that the MOH has prioritized before we can think of deworming,” said Ms. Kitenge.	

*Vignette 3: Low change commitment, high change efficacy (moderate overall readiness)*

**Table:**

In Bahari, a fictional country in East Africa, many people are infected with intestinal worms. As recommended by the World Health Organization, the country’s focus has been on administering deworming medications to children in primary schools. However, as a result, many adolescents and adults have not been dewormed. The number of worm infections in Bahari is not decreasing and government officials believe it may be due to ongoing infections in these older age groups. The Ministry of Health (MOH) has decided that it will launch a new community-wide deworming campaign to make sure that everyone in the population—children, adolescents, and adults—is dewormed. The government’s goal is to stop the spread of intestinal worm infections and to improve the overall health of the community.	
Jane Daktari, the director of MOH in Bahari, expressed hesitancy that the community-wide campaign will be successful. She said, “How do we know that providing deworming to all of the members in our community will be effective?” Mary Kitenge, a county-level MOH office, added, “Local health system personnel have their own concerns. We will try our best to make sure that there is high coverage in all targeted villages but it will be difficult because people will not feel interested in dealing with worms. I’m not sure there is buy-in at the local level.” John Kanga, the Neglected Tropical Disease Programme Manager, said, “We have been implementing the school deworming campaign for several years now and will use that experience to make sure the community-wide campaign is a success. We have the necessary resources, as the national MOH has allocated funding in its annual budget to include the community-wide deworming campaign and we have a large number of community health workers who can administer the deworming medicines.” Despite her concerns, Daktari added, “This is a high priority campaign for the Ministry so we will be sure to carefully schedule the campaign until after the rainy season for ease of implementation,” said Daktari.	

*Vignette 4: Low change commitment, low change efficacy (low overall readiness)*

**Table:**

In Bahari, a fictional country in East Africa, many people are infected with intestinal worms. As recommended by the World Health Organization, the country’s focus has been on administering deworming medications to children in primary schools. However, as a result, many adolescents and adults have not been dewormed. The number of worm infections in Bahari is not decreasing and government officials believe it may be due to ongoing infections in these older age groups. The Ministry of Health (MOH) has decided that it will launch a new community-wide deworming campaign to make sure that everyone in the population—children, adolescents, and adults—is dewormed. The government’s goal is to stop the spread of intestinal worm infections and to improve the overall health of the community.	
Jane Daktari, the director of MOH in Bahari, expressed hesitancy that the community-wide campaign will be successful. She said, “How do we know that providing deworming to all of the members in our community will be effective? We should be focusing on interventions that improve water and sanitation and not just deworming alone.” Mary Kitenge, a County-level MOH office, added, “Local health system personnel have their own concerns. We will try our best to make sure that there is high coverage in all targeted villages but it will be difficult because people will not feel interested in dealing with worms. I’m not sure there is buy-in at the local level.” John Kanga, the Neglected Tropical Disease Programme Manager in Bahari said, “We have been relying on technical partners to implement the school deworming campaign for several years now. We at MOH will have to learn how to do the community-wide campaign on our own so that we can make sure it is a success. That could be challenging” Mr. Kanga will be transferred to a different department within the Ministry soon and there will be a new NTD manager who needs to manage the campaign. “The first thing this person will need to do is ensure that we have all of the necessary resources to make sure that everyone is reached. The national MOH has not allocated funding in its annual budget to include the community-wide deworming campaign. Also, I am concerned that we do not have enough deworming medicines in the country for such a large-scale campaign,” said Mr. Kanga. Ms. Kitenge added, “We do not have enough existing community health workers who are ready to begin administration. Even if we hire enough, we will first need to focus on the immunization campaign that the MOH has prioritized before we can think of deworming,” said Ms. Kitenge.	

### Appendix 2: Survey questions by construct, sub-construct and domains

**Table 6:** Descriptive items in the structural readiness survey, including response options, by country

Question	Benin	India	Malawi
At what level do you work?	• National • Department • Commune • Health center • Village (drug distributor) • World Health Organization • Implementation partner	• National • State • District • Sub-district • Block • Drug distributor/ASHA • World health organization • Implementation partner	• National • Zone/region • District • Community-health center • Community-health surveillance assistant • World Health Organization • Implementation partner
Does [COUNTRY] have a national policy for the control of neglected tropical diseases (NTDs)?	Yes/no		
Does [COUNTRY] have an NTD Master Plan?	Yes/no		
If yes, what is the STH-specific strategy in the NTD Master Plan?	• Deworming of children (ages 2–14 years) who are both enrolled in school and not enrolled in school. • Deworming of children (ages 5–14 years) who are enrolled in schools. • Deworming of children (ages 2–14 years) who are enrolled in schools. • Deworming of children (ages 5–14 years) who are both enrolled in school and not enrolled in school.	• National deworming day once per year during which pre-school and school-age children are dewormed at school. • National deworming day every 6 months, during which pre-school and school-age children are dewormed at school. • National deworming day every 6 months during which all members of the community are dewormed in their homes. • Other (please specify)	• National deworming day once per year during which pre-school and school-age children are dewormed at school. • National deworming day every 6 months, during which pre-school and school-age children are dewormed at school. • National deworming day every 6 months during which all members of the community are dewormed in their homes. • Other (please specify)
[BENIN/INDIA] Have you worked as a drug distributor for other community-based MDA programs before? [MALAWI] Have you worked as a Health Surveillance Assistants (HSA) for other community-based MDA programmes before?	Yes/no		
How often do you present new ideas to your supervisor?	Never, rarely, occasionally, often, always, not enough information to answer		
What is the exact amount of payment or other incentives provided to drug distributors in [COUNTRY] when implementing a community-based programme?	• 500 CFA per day • 1000 CFA per day • 2000 CFA per day • 3000 CFA per day	Open-ended	
Please select 3 materials that you think are most key to delivering community-wide MDA (besides deworming medicines)	• IEC materials (for community sensitization) • Well-trained staff • Access to printing for field materials • Incentives for volunteer drug distribution • Vehicles and fuel • MDA delivery guide for volunteer drug distributors • Other		• IEC materials (for community sensitization) • Well-trained staff • Access to printing for field materials • Incentives for Health Surveillance Assistants (HSA) • Vehicles and fuel • MDA delivery guide for Health Surveillance Assistants (HSA) • Other (please specify)
[BENIN] How many drug distributors are typically needed to deliver MDA in a community of 5,000 people? [MALAWI/INDIA] How many Health Surveillance Assistants are typically needed to deliver MDA in a community of 5,000 people?	Insert number		
[BENIN/INDIA] I am involved in the training, implementation or supervision of NTD program research activities in [COUNTRY]. [MALAWI] I am involved in training or supervision of HSA in [COUNTRY].	Yes/no		
[BENIN/INDIA] I regularly work with drug distributors in my current job capacity. [MALAWI] I regularly work with HSAs in my current job capacity.	Never, rarely, occasionally, often, always, not enough information to answer		
I regularly oversee MDA activities at a local/community level in my current job capacity.	Never, rarely, occasionally, often, always, not enough information to answer		

**Table 7:** Structural readiness items by construct, sub-construct and domains

Construct	Sub-construct	Item	Domains
**Change commitment**	*Needed*	I believe that [COUNTRY] needs to interrupt STH transmission.	Policy environment
		I have observed that my co-workers generally believe that [COUNTRY] needs to interrupt STH transmission.	Policy environment
	*Motivated*	I am supportive of implementing community-wide MDA for STH.	Policy environment
		I have observed that my co-workers are generally supportive of implementing community-wide MDA for STH.	Policy environment
		Ministry of Education personnel that I work with on school or child interventions will likely support transitioning from school-based to community-wide deworming.	Leadership
		In my experience, community drug distributors are given sufficient financial and/or non-financial incentives for administering community-wide MDA.	Financial resources
	*Outcome expectancy*	I believe that community-wide MDA can interrupt STH transmission in [COUNTRY].*	Policy environment
		I have observed that my co-workers generally believe that community-wide MDA can interrupt STH transmission in [COUNTRY].	Policy environment
**Change efficacy**	*Task demand*	In my experience, community drug distributor supervisors provide good guidance to distributors on how to deliver community-wide MDA.	Leadership
		[Stakeholder level] staff will need additional training to effectively deliver community-wide MDA for STH.	Human resources
		Additional supervisors are needed at [stakeholder level] to coordinate the delivery of community-wide MDA for STH.	Human resources
		In my experience, personnel at [stakeholder level] have demonstrated that they can deliver other community-wide MDA programmes (ex. lymphatic filariasis) with high coverage.	Technical capacity
	*Resource availability*	How often have you observed difficulties with having enough funding at the National level to support implementation of community-based programmes?	Financial resources
		How often do you encounter difficulties with having enough funding at the district level to implement of community-based programmes?	Financial resources
		I am not worried about whether [COUNTRY] has sufficient future funding for community-wide MDA programmes.	Financial resources
		[COUNTRY] currently has the resources and tools needed to develop high-quality sensitization and education materials for community-wide MDA for STH.	Material resources
		In my experience, there is an effective programme in [COUNTRY] for training community drug distributors on how to deliver community-wide MDA.	Technical capacity
		I know of at least one community health programme that could be used to deliver community-wide MDA for STH.*	Community delivery-infrastructure
	*Contextual factors*	I have observed that there is a collaborative network of external stakeholders (NGOs or technical/financial partners) that would support community-wide MDA for STH in [COUNTRY].	Policy environment
		How often are community members in [COUNTRY] resistant to community-wide MDA campaigns?	Community-delivery infrastructure
**Capacity**	*Demonstrated capacity*	In my experience, [COUNTRY’s] National NTD Master Plan is currently being implemented as intended.	Policy environment
		How often have you encountered difficulty moving money across [stakeholder level of the health system] for a community-based programme?	Financial resources
		How often have you observed delays in the arrival of drugs for MDA programmes due to supply chain problems?	Material capacity
		I have observed that it is challenging to recruit enough community drug distributors needed in [COUNTRY] to deliver community-wide MDA.	Human resources
		How often are treatment data incorrectly recorded during delivery of community-wide MDA programmes?	Technical capacity
		In my experience, community drug distributors have the skills to effectively deliver community-wide MDA for STH.	Community-delivery infrastructure
**Flexibility**	*Flexibility*	It is challenging to present new ideas to my supervisor.	Leadership
		In my experience, when MOH leadership at the National level are presented with new ideas, research activities, or pilot projects, they are generally receptive to them.	Leadership
		How often do your **supervisors** generally feel comfortable **receiving** feedback and recommendations from you and your colleagues on how to improve the delivery of interventions?	Leadership
		How often do your **subordinates** generally feel comfortable **providing** feedback and recommendations on how to improve the delivery of interventions?	Leadership
**Organizational structure**	*Leadership structure*	In my experience, the NTD programme leadership at the national level is effectively implementing community-wide MDA programmes in [COUNTRY].	Leadership
		In my experience, the NTD programme leadership at the [STATE] level is effectively implementing NTD programmes in [COUNTRY].	Leadership
		In my experience, the NTD programme leadership at the [DISTRICT]l level is effectively implementing NTD programmes in [COUNTRY].	Leadership
	*Political structure*	In my experience, [COUNTRY] 's national policy for NTD control supports community-wide MDA.	Policy environment
		I have observed that [COUNTRY]'s National NTD Master Plan provides sufficient guidance for delivering community-wide MDA programmes, such as lymphatic filariasis (LF).	Policy environment
		I have observed that deworming medicines are acquired centrally and re-distributed to local levels without too much difficulty.	Material resources

Likert scale 1–5: 5 being most positive, 1 being most negative. Most response options are coded as 1 (Disagree or Never), 2 (Somewhat disagree or Rarely), 3 (Unsure or Occasionally), 4 (Somewhat agree or Often), and 5 (Agree or Always), with an option of “Not enough information to answer” if necessary. Some response options are necessarily reverse coded.

### Appendix 3: Item-level median and interquartile range responses for each stakeholder level and country

**Table 8:** Item-level median and interquartile ranges in Benin (n=122)

Construct	Sub-construct	Item	Policymakers (n=15)	Mid-level managers (n=9)	Implementers (n=98)
**Change commitment**	*Needed*	I believe that Benin needs to interrupt STH transmission.	4.0 (1.0)	5.0 (0)	5.0 (1.0)
		I have observed that my co-workers generally believe that Benin needs to interrupt STH transmission.	4.0 (2.0)	5.0 (1.0)	5.0 (1.0)
		***Needed median***	**4.0 (1.0)**	**5.0 (0.5)**	**5.0 (1.0)**
	*Motivated*	I am supportive of implementing community-wide MDA for STH.	5.0 (2.0)	5.0 (0)	5.0 (1.0)
		I have observed that my co-workers are generally supportive of implementing community-wide MDA for STH.	5.0 (2.0)	4.0 (2.0)	5.0 (1.0)
		Ministry of Education personnel that I work with on school or child interventions will likely support transitioning from school-based to community-wide deworming.	5.0 (1.0)	4.0 (1.0)	5.0 (1.0)
		In my experience, community drug distributors are given sufficient financial and/or non-financial incentives for administering community-wide MDA.	3.0 (2.0)	2.0 (3.0)	4.0 (2.0)
		***Motivated median***	**4.5 (1.5)**	**3.5 (2.0)**	**4.5 (1.0)**
	*Outcome expectancy*	I believe that community-wide MDA can interrupt STH transmission in Benin.*	4.0 (2.0)	5.0 (0)	5.0 (1.0)
		I have observed that my co-workers generally believe that community-wide MDA can interrupt STH transmission in Benin.	4.0 (2.0)	4.0 (2.0)	5.0 (1.0)
		***Outcome expectancy median***	**4.0 (2.0)**	**4.5 (1.0)**	**4.5 (1.5)**
		**Change commitment median**	**4.0 (2.0)**	**4.5 (1.0)**	**5.0 (1.0)**
**Change efficacy**	*Task demand*	In my experience, community drug distributor supervisors provide good guidance to distributors on how to deliver community-wide MDA.			5.0 (1.0)^b^
		[Stakeholder level] staff will need additional training to effectively deliver community-wide MDA for STH.	3.0 (3.0)^a^	1.0 (0)	1.0 (1.0)
		Additional supervisors are needed at [stakeholder level] to coordinate the delivery of community-wide MDA for STH.	2.0 (0)^a^	2.0 (2.0)	1.0 (1.0)
		In my experience, personnel at [stakeholder level] have demonstrated that they can deliver other community-wide MDA programmes (ex. lymphatic filariasis) with high coverage.	5.0 (1.0)	4.0 (1.0)	4.0 (2.0)
		***Task demand median***	**4.0 (2.0)**	**2.0 (2.0)**	**1.0 (2.0)**
	*Resource availability*	How often have you observed difficulties with having enough funding at the National level to support implementation of community-based programmes?	3.0 (2.0)	3.0 (2.0)	3.0 (1.0)^b^
		How often do you encounter difficulties with having enough funding at the district level to implement of community-based programmes?	2.0 (1.0)	4.0 (2.0)	3.0 (2.0)
		I am not worried about whether Benin has sufficient future funding for community-wide MDA programmes.	2.0 (1.0)^c^	3.0 (1.0)	
		Benin currently has the resources and tools needed to develop high-quality sensitization and education materials for community-wide MDA for STH.	5.0 (2.0)	3.0 (2.0)	5.0 (2.0)
		In my experience, there is an effective programme in Benin for training community drug distributors on how to deliver community-wide MDA.	5.0 (1.0)	3.0 (3.0)	5.0 (1.0)
		I know of at least one community health programme that could be used to deliver community-wide MDA for STH.*	5.0 (2.0)	5.0 (2.0)	4.0 (2.0)
		***Resource availability median***	**3.5 (1.5)**	**3.0 (1.0)**	**4.0 (1.5)**
	*Contextual factors*	I have observed that there is a collaborative network of external stakeholders (NGOs or technical/financial partners) that would support community-wide MDA for STH in Benin.	4.0 (2.0)	3.0 (4.0)	5.0 (1.0)
		How often are community members in [country] resistant to community-wide MDA campaigns?	4.0 (1.0)	2.0 (2.0)	3.0 (2.0)
		***Contextual factors median***	**4.0 (0.5)**	**3.0 (3.0)**	**3.8 (1.0)**
		**Change efficacy median**	**4.0 (1.0)**	**3.0 (1.0)**	**4.0 (1.0)**
**Organizational readiness for change**			**4.0 (1.0)**	**3.0 (1.0)**	**4.0 (1.0)**
**Capacity**	*Demonstrated capacity*	In my experience, Benin’s National NTD Master Plan is currently being implemented as intended.	5.0 (2.0)^a^	2.0 (1.0)	
		How often have you encountered difficulty moving money across [stakeholder level of the health system] for a community-based programme?	3.0 (0)^c^	3.0 (2.0)	3.0 (1.0)^b^
		How often have you observed delays in the arrival of drugs for MDA programmes due to supply chain problems?	4.0 (2.0)	3.0 (2.0)	4.0 (1.0)
		I have observed that it is challenging to recruit enough community drug distributors needed in Benin to deliver community-wide MDA.	4.0 (1.0)	4.0 (2.0)	2.5 (3.0)
		How often are treatment data incorrectly recorded during delivery of community-wide MDA programmes?	3.0 (0)	3.0 (2.0)	3.0 (2.0)
		In my experience, community drug distributors have the skills to effectively deliver community-wide MDA for STH.	4.0 (1.0)	4.0 (3.0)	4.0 (2.0)^b^
		**Capacity median**	**3.5 (1.0)**	**3.0 (2.0)**	**3.0 (2.0)**
**Flexibility**	*Flexibility*	It is challenging to present new ideas to my supervisor.	4.0 (2.0)^c^	2.0 (2.0)	
		In my experience, when MOH leadership at the National level are presented with new ideas, research activities, or pilot projects, they are generally receptive to them.	4.0 (2.0)^a^	3.0 (0)	
		How often do your **supervisors** generally feel comfortable **receiving** feedback and recommendations from you and your colleagues on how to improve the delivery of interventions?	4.0 (1.0)^c^	3.0 (1.0)	
		How often do your **subordinates** generally feel comfortable **providing** feedback and recommendations on how to improve the delivery of interventions?	4.0 (1.0)^c^	4.0 (2.0)	
		**Flexibility median**	**4.0 (2.0)**^**a**^	**3.5 (0.5)**	
**Organizational structure**	*Leadership structure*	In my experience, the NTD programme leadership at the national level is effectively implementing community-wide MDA programmes in Benin.	5.0 (1.0)^a^	4.0 (1.0)	
		In my experience, the NTD programme leadership at the [STATE] level is effectively implementing NTD programmes in Benin.	5.0 (1.0)^a^	2.0 (1.0)	
		In my experience, the NTD programme leadership at the [DISTRICT] level is effectively implementing NTD programmes in Benin.	5.0 (2.0)^a^	2.0 (1.0)	
		***Leadership structure median***	**5.0 (1.0)**^**a**^	**2.0 (2.0)**	
	*Political structure*	In my experience, Benin's national policy for NTD control supports community-wide MDA.	5.0 (0)^a^		
		I have observed that Benin's National NTD Master Plan provides sufficient guidance for delivering community-wide MDA programmes, such as lymphatic filariasis (LF).	5.0 (2.0)^a^	2.0 (1.0)	
		I have observed that deworming medicines are acquired centrally and re-distributed to local levels without too much difficulty.	5.0 (1.0)	4.0 (2.0)	3.5 (3.5)^b^
		***Political structure median***	**5.0 (1.0)**	**3.5 (1.0)**	**3.5 (3.5)**^**b**^
		**Organizational structure median**	**5.0 (1.0)**	**2.0 (2.0)**	**3.5 (3.5)**
	**Organizational capacity for change**		**4.0 (1.0)**	**3.0 (1.0)**	**3.0 (2.0)**
	**Summary item**: I believe that Benin is ready to implement community-wide MDA for STH for the first time		4.0 (2.0)	3.0 (2.0)	5.0 (2.0)

^a^Implementation partners not asked the question

^b^CDDs not asked the question (only health centers)

^c^Implementation partners and WHO not asked the question

**Table 9:** Item-level median and interquartile ranges in India (n = 74)

Construct	Sub-construct	Item	Policymakers N=3	Mid-level managers N=5	Implementers N=66
**Change commitment**	*Needed*	I believe that India needs to interrupt STH transmission.	5.0 (0)	5.0 (0)	5.0 (0)
		I have observed that my co-workers generally believe that India needs to interrupt STH transmission.	5.0 (0)	5.0 (1.0)	5.0 (0)
		***Needed median***	**5.0 (0)**	**5.0 (0.5)**	**5.0 (0)**
	*Motivated*	I am supportive of implementing community-wide MDA for STH.	5.0 (0)	5.0 (0)	5.0 (0)
		I have observed that my co-workers are generally supportive of implementing community-wide MDA for STH.	5.0 (0)	5.0 (1.0)	5.0 (0)
		Ministry of Education personnel that I work with on school or child interventions will likely support transitioning from school-based to community-wide deworming.	4.0 (2.0)	5.0 (0)	5.0 (0)
		In my experience, community drug distributors are given sufficient financial and/or non-financial incentives for administering community-wide MDA.	5.0 (3.0)	5.0 (2.0)	4.0 (3.0)
		***Motivated median***	**5.0 (0)**	**5.0 (0)**	**5.0 (0)**
	*Outcome expectancy*	I believe that community-wide MDA can interrupt STH transmission in India.	5.0 (0)	5.0 (0)	5.0 (0)
		I have observed that my co-workers generally believe that community-wide MDA can interrupt STH transmission in India.	5.0 (0)	5.0 (1.0)	5.0 (1.0)
		***Outcome expectancy median***	**5.0 (0)**	**5.0 (0.5)**	**5.0 (0.5)**
		**Change commitment median**	**5.0 (0)**	**5.0 (0)**	**5.0 (0)**
**Change efficacy**	*Task demand*	In my experience, community drug distributor supervisors provide good guidance to distributors on how to deliver community-wide MDA.			5.0 (0.5)
		[Stakeholder level] staff will need additional training to effectively deliver community-wide MDA for STH.	4.0 (1.0)^a^	3.0 (1.0)	1.0 (1.0)
		Additional supervisors are needed at [stakeholder level] to coordinate the delivery of community-wide MDA for STH.	1.0 (3.0)^a^	2.0 (1.0)	1.0 (3.0)
		In my experience, personnel at [stakeholder level] have demonstrated that they can deliver other community-wide MDA programmes (ex. lymphatic filariasis) with high coverage.	5.0 (2.0)	5.0 (0)	5.0 (1.0)
		***Task demand median***	**4.0 (1.0)**	**2.0 (1.0)**	**3.0 (2.0)**
	*Resource availability*	How often have you observed difficulties with having enough funding at the National level to support implementation of community-based programmes?	3.0 (2.0)	4.0 (0)	3.0 (2.0)^b^
		How often do you encounter difficulties with having enough funding at the district level to implement of community-based programmes?	2.0 (3.0)	4.0 (0)	3.0 (2.0)
		I am not worried about whether India has sufficient future funding for community-wide MDA programmes.	3.0 (4.0)^c^	3.0 (2.0)	
		India currently has the resources and tools needed to develop high-quality sensitization and education materials for community-wide MDA for STH.	5.0 (3.0)	4.0 (0)	5.0 (2.0)
		In my experience, there is an effective programme in India for training community drug distributors on how to deliver community-wide MDA.	4.0 (2.0)	4.0 (1.0)	4.5 (2.0)
		I know of at least one community health programme that could be used to deliver community-wide MDA for STH.	5.0 (1.0)	4.0 (1.0)	5.0 (2.0)
		***Resource availability median***	**4.0 (3.0)**	**4.0 (0)**	**4.5 (1.5)**
	*Contextual factors*	I have observed that there is a collaborative network of external stakeholders (NGOs or technical/financial partners) that would support community-wide MDA for STH in India.	5.0 (0)	5.0 (0)	5.0 (1.0)
		How often are community members in India resistant to community-wide MDA campaigns?	4.0 (2.0)	4.0 (2.0)	5.0 (1.0)
		***Contextual factors median***	**4.5 (1.0)**	**4.5 (1.0)**	**4.5 (1.0)**
		**Change efficacy median**	**4.0 (2.5)**	**4.0 (0)**	**4.5 (1.5)**
**Organizational readiness for change**			**5.0 (0.5)**	**4.0 (1.0)**	**5.0 (0.5)**
**Capacity**	*Demonstrated capacity*	In my experience, India‘s National NTD Master Plan is currently being implemented as intended.	3.0 (2.0)	3.0 (2.0)	
		How often have you encountered difficulty moving money across [stakeholder level of the health system] for a community-based programme?	3.5 (1.0)^c^	4.0 (0)	3.0 (3.0)^b^
		How often have you observed delays in the arrival of drugs for MDA programmes due to supply chain problems?	2.0 (1.0)	5.0 (1.0)	5.0 (2.0)
		I have observed that it is challenging to recruit enough community drug distributors needed in India to deliver community-wide MDA.	1.0 (2.0)	2.0 (0)	3.0 (4.0)
		How often are treatment data incorrectly recorded during delivery of community-wide MDA programmes?	3.0 (1.0)	4.0 (1.0)	4.5 (2.0)
		In my experience, community drug distributors have the skills to effectively deliver community-wide MDA for STH.	3.0 (2.0)	5.0 (0)	5.0 (1.0)^b^
		***Demonstrated capacity median***	**3.0 (1.0)**	**4.5 (1.0)**	**5.0 (2.0)**
**Flexibility**	Flexibility	It is challenging to present new ideas to my supervisor.	1.0 (0)^c^	4.0 (1.0)	
		In my experience, when MOH leadership at the National level are presented with new ideas, research activities, or pilot projects, they are generally receptive to them.	5.0 (0)^a^	4.0 (1.0)	
		How often do your **supervisors** generally feel comfortable **receiving** feedback and recommendations from you and your colleagues on how to improve the delivery of interventions?	5.0 (0)^c^	5.0 (2.0)	
		How often do your **subordinates** generally feel comfortable **providing** feedback and recommendations on how to improve the delivery of interventions?	5.0 (0)^c^	5.0 (0)	
		**Flexibility median**	**5.0 (0)**	**4.5 (0.5)**	
**Organizational structure**	*Leadership structure*	In my experience, the NTD programme leadership at the national level is effectively implementing community-wide MDA programmes in India.	5.0 (0)^a^	5.0 (2.0)	
		In my experience, the NTD programme leadership at the [STATE] level is effectively implementing NTD programmes in India.	4.0 (1.0)^a^	5.0 (2.0)	
		In my experience, the NTD programme leadership at the [DISTRICT]l level is effectively implementing NTD programmes in India.	4.0 (1.0)^a^	5.0 (1.0)	
		***Leadership structure median***	**4.0 (1.0)**	**5.0 (2.0)**	
	*Political structure*	In my experience, India's national policy for NTD control supports community-wide MDA.	5.0 (2.0)^a^		
		I have observed that India’s National NTD Master Plan provides sufficient guidance for delivering community-wide MDA programmes, such as lymphatic filariasis (LF).	4.0 (2.0)^a^	3.0 (2.0)	
		I have observed that deworming medicines are acquired centrally and re-distributed to local levels without too much difficulty.	4.0 (3.0)	5.0 (0)	5.0 (0)^b^
	***Political structure median***		**5.0 (2.0)**	**4.0 (1.0)**	**5.0 (0)**
	**Organizational structure median**		**4.5 (1.5)**	**5.0 (2.0)**	**5.0 (0)**
**Organizational capacity for change**			**4.5 (1.0)**	**4.5 (1.0)**	**5.0 (1.5)**
**Summary item**: I believe that India is ready to implement community-wide MDA for STH for the first time			3.0 (1.0)	5.0 (0)	5.0 (1.0)

^a^Implementation partners not asked the question

^b^CDDs not asked the question (only health centers)

^c^Implementation partners and WHO not asked the question

**Table 10:** Item-level median and interquartile ranges in Malawi (n = 79)

Construct	Sub-construct	Item	Policymakers (n=12)	Mid-level managers (n=10)	Implementers (n=57)
**Change commitment**	*Needed*	I believe that Malawi needs to interrupt STH transmission.	5.0 (0)	5.0 (0)	5.0 (0)
		I have observed that my co-workers generally believe that Malawi needs to interrupt STH transmission.	5.0 (0)	5.0 (0)	5.0 (0)
		***Needed median***	**5.0 (0)**	**5.0 (0)**	**5.0 (0)**
	*Motivated*	I am supportive of implementing community-wide MDA for STH.	5.0 (0)	5.0 (0)	5.0 (0)
		I have observed that my co-workers are generally supportive of implementing community-wide MDA for STH.	5.0 (0)	5.0 (0)	5.0 (0)
		Ministry of Education personnel that I work with on school or child interventions will likely support transitioning from school-based to community-wide deworming.	5.0 (0)	5.0 (0)	5.0 (1.0)
		In my experience, community drug distributors are given sufficient financial and/or non-financial incentives for administering community-wide MDA.	4.5 (1.0)	2.0 (2.0)	1.0 (1.0)
		***Motivated median***	**5.0 (0.25)**	**5.0 (0)**	**5.0 (0.5)**
	*Outcome expectancy*	I believe that community-wide MDA can interrupt STH transmission in Malawi.	5.0 (0.5)	5.0 (0)	5.0 (0)
		I have observed that my co-workers generally believe that community-wide MDA can interrupt STH transmission in Malawi	5.0 (1.0)	5.0 (0)	5.0 (0)
		***Outcome expectancy median***	**4.8 (0.5)**	**5.0 (0)**	**5.0 (0)**
		**Change commitment median**	**5.0 (0)**	**5.0 (0)**	**5.0 (0)**
**Change efficacy**	*Task demand*	In my experience, community drug distributor supervisors provide good guidance to distributors on how to deliver community-wide MDA.			5.0 (0)
		[Stakeholder level] staff will need additional training to effectively deliver community-wide MDA for STH.	2.0 (1.0) ^a^	1.5 (1.0)	1.0 (0)
		Additional supervisors are needed at [stakeholder level] to coordinate the delivery of community-wide MDA for STH.	1.5 (3.0) ^a^	3.0 (3.0)	1.0 (4.0)
		In my experience, personnel at [stakeholder level] have demonstrated that they can deliver other community-wide MDA programmes (ex. lymphatic filariasis) with high coverage.	5.0 (0)	5.0 (1.0)	5.0 (1.0)
		***Task demand median***	**4.0 (3.5)**	**3.0 (3.0)**	**3.0 (1.5)**
	*Resource availability*	How often have you observed difficulties with having enough funding at the National level to support implementation of community-based programmes?	3.0 (1.0)^c^	3.0 (2.0)	2.0 (1.0)^b^
		How often do you encounter difficulties with having enough funding at the district level to implement of community-based programmes?	3.0 (2.0)	2.5 (2.0)	3.0 (1.0)
		I am not worried about whether Malawi has sufficient future funding for community-wide MDA programmes.	2.5 (4.0) ^a^	3.0 (1.0)	
		Malawi currently has the resources and tools needed to develop high-quality sensitization and education materials for community-wide MDA for STH.	3.5 (3.0)	4.0 (4.0)	3.0 (3.0)
		In my experience, there is an effective programme in Malawi for training community drug distributors on how to deliver community-wide MDA.	4.0 (2.0)	5.0 (1.0)	4.0 (3.0)
		I know of at least one community health programme that could be used to deliver community-wide MDA for STH.*	5.0 (0)	5.0 (1.0)	4.0 (2.0)
	***Resource availability median***		**3.5 (1.0)**	**3.5 (1.0)**	**3.0 (1.5)**
	*Contextual factors*	I have observed that there is a collaborative network of external stakeholders (NGOs or technical/financial partners) that would support community-wide MDA for STH in Malawi.	5.0 (1.0)	5.0 (0)	5.0 (2.0)
		How often are community members in Malawi resistant to community-wide MDA campaigns?	3.5 (1.0)	3.0 (0)	3.0 (2.0)
		***Contextual factors median***	**4.0 (0.8)**	**4.0 (0.5)**	**4.0 (1.5)**
		**Change efficacy median**	**4.0 (0)**	**3.0 (1.0)**	**3.5 (1.0)**
	**Organizational readiness for change**		**5.0 (1.0)**	**5.0 (1.0)**	**5.0 (1.0)**
**Capacity**	*Demonstrated capacity*	In my experience, Malawi’s National NTD Master Plan is currently being implemented as intended.	5.0 (1.0)	5.0 (3.0)	
		How often have you encountered difficulty moving money across [stakeholder level of the health system] for a community-based programme?	3.0 (1.5)^c^	3.0 (2.0)	3.0 (0) ^b^
		How often have you observed delays in the arrival of drugs for MDA programmes due to supply chain problems?	3.5 (1.0)	3.0 (1.0)	4.0 (2.0)
		I have observed that it is challenging to recruit enough community drug distributors needed in Malawi to deliver community-wide MDA.	4.0 (2.5)	3.5 (3.0)	2.0 (3.0)
		How often are treatment data incorrectly recorded during delivery of community-wide MDA programmes?	3.0 (1.5)	3.5 (1.0)	4.0 (0)
		In my experience, community drug distributors have the skills to effectively deliver community-wide MDA for STH.	5.0 (0)	5.0 (1.0)	*5.0 (0)* ^b^
		**Capacity median**	**3.5 (0.8)**	**3.8 (1.0)**	**4.0 (1.0)**
**Flexibility**	*Flexibility*	It is challenging to present new ideas to my supervisor.	3.5(1.0)^c^	3.5 (1.0)	
		In my experience, when MOH leadership at the National level are presented with new ideas, research activities, or pilot projects, they are generally receptive to them.	5.0 (0)^a^	4.0 (3.0)	
		How often do your **supervisors** generally feel comfortable **receiving** feedback and recommendations from you and your colleagues on how to improve the delivery of interventions?	3.0 (0.5) ^c^	2.5 (2.0)	
		How often do your **subordinates** generally feel comfortable **providing** feedback and recommendations on how to improve the delivery of interventions?	4.0 (2.0)^c^	4.0 (1.0)	
		**Flexibility median**	**3.5 (1.5)**	**3.3 (1.5)**	
**Organizational structure**	*Leadership structure*	In my experience, the NTD programme leadership at the national level is effectively implementing community-wide MDA programmes in Malawi.	5.0 (0.5) ^a^	4.5 (2.0)	
		In my experience, the NTD programme leadership at the [STATE] level is effectively implementing NTD programmes in Malawi.	3.5 (4.0) ^a^	4.5 (3.0)	
		In my experience, the NTD programme leadership at the [DISTRICT] level is effectively implementing NTD programmes in Malawi.	5.0 (0.5) ^a^	4.0 (2.0)	
		***Leadership structure median***	**5.0 (0.5)**	**4.5 (2.0)**	
	*Political structure*	In my experience, Malawi's national policy for NTD control supports community-wide MDA.	5.0 (0) ^a^		
		I have observed that Malawi's National NTD Master Plan provides sufficient guidance for delivering community-wide MDA programmes, such as lymphatic filariasis (LF).	5.0 (1.0)*	5.0 (2.0)	
		I have observed that deworming medicines are acquired centrally and re-distributed to local levels without too much difficulty.	4.5 (2.0)	4.0 (2.0)	5.0 (2.0) ^b^
		***Political structure median***	**5.0 (1.8)**	**4.3 (2.5)**	**5.0 (2.0)**
		**Organizational structure median**	**4.5 (1.8)**	**4.5 (2.0)**	**5.0 (1.0)**
		**Organizational capacity for change**	**4.0 (0.5)**	**3.5 (1.0)**	**4.0 (1.5)**
**Summary item**: I believe that Malawi is ready to implement community-wide MDA for STH for the first time			5.0 (0.5)	5.0 (0)	5.0 (1.0)

^a^Implementation partners not asked the question

^b^CDDs not asked the question (only health centers)

^c^Implementation partners and WHO not asked the question

### Appendix 4

**Table 11:** Item level codes corresponding to Figs. 2, 3, and 4

Construct	Sub-construct	Item	Figure code
**Change commitment**	*Needed*	I believe that *Country* needs to interrupt STH transmission.	CC1
		I have observed that my co-workers generally believe that *Country* needs to interrupt STH transmission.	CC2
	*Motivated*	I am supportive of implementing community-wide MDA for STH.	CC3
		I have observed that my co-workers are generally supportive of implementing community-wide MDA for STH.	CC4
		Ministry of Education personnel that I work with on school or child interventions will likely support transitioning from school-based to community-wide deworming.	CC5
		In my experience, community drug distributors are given sufficient financial and/or non-financial incentives for administering community-wide MDA.	CC6
	*Outcome expectancy*	I believe that community-wide MDA can interrupt STH transmission in *Country*.	CC7
		I have observed that my co-workers generally believe that community-wide MDA can interrupt STH transmission in *Country*	CC8
**Change efficacy**	*Task demand*	In my experience, community drug distributor supervisors provide good guidance to distributors on how to deliver community-wide MDA.	CE
		[Stakeholder level] staff will need additional training to effectively deliver community-wide MDA for STH.	CE2
		Additional supervisors are needed at [stakeholder level] to coordinate the delivery of community-wide MDA for STH.	CE3
		In my experience, personnel at [stakeholder level] have demonstrated that they can deliver other community-wide MDA programmes (ex. lymphatic filariasis) with high coverage.	CE4
	*Resource availability*	How often have you observed difficulties with having enough funding at the National level to support implementation of community-based programmes?	CE5
		How often do you encounter difficulties with having enough funding at the district level to implement of community-based programmes?	CE6
		I am not worried about whether *Country* has sufficient future funding for community-wide MDA programmes.	CE7
		*Country* currently has the resources and tools needed to develop high-quality sensitization and education materials for community-wide MDA for STH.	CE8
		In my experience, there is an effective programme in *Country* for training community drug distributors on how to deliver community-wide MDA.	CE9
		I know of at least one community health programme that could be used to deliver community-wide MDA for STH.*	CE10
	*Contextual factors*	I have observed that there is a collaborative network of external stakeholders (NGOs or technical/financial partners) that would support community-wide MDA for STH in *Country*.	CE11
		How often are community members in Malawi resistant to community-wide MDA campaigns? *Country*	CE12
**Capacity**	*Demonstrated capacity*	In my experience, *Country* National NTD Master Plan is currently being implemented as intended.	CAP1
		How often have you encountered difficulty moving money across [stakeholder level of the health system] for a community-based programme?	CAP2
		How often have you observed delays in the arrival of drugs for MDA programmes due to supply chain problems?	CAP3
		I have observed that it is challenging to recruit enough community drug distributors needed in *Country* to deliver community-wide MDA.	CAP4
		How often are treatment data incorrectly recorded during delivery of community-wide MDA programmes?	CAP5
		In my experience, community drug distributors have the skills to effectively deliver community-wide MDA for STH?	CAP6
**Flexibility**	*Flexibility*	It is challenging to present new ideas to my supervisor.	FLEX1
		In my experience, when MOH leadership at the National level are presented with new ideas, research activities, or pilot projects, they are generally receptive to them.	FLEX2
		How often do your **supervisors** generally feel comfortable **receiving** feedback and recommendations from you and your colleagues on how to improve the delivery of interventions?	FLEX3
		How often do your **subordinates** generally feel comfortable **providing** feedback and recommendations on how to improve the delivery of interventions?	FLEX4
**Organizational structure**	*Leadership structure*	In my experience, the NTD programme leadership at the national level is effectively implementing community-wide MDA programmes in *Country*.	ORG1
		In my experience, the NTD programme leadership at the [STATE] level is effectively implementing NTD programmes in *Country*.	ORG2
		In my experience, the NTD programme leadership at the [DISTRICT] level is effectively implementing NTD programmes in *Country*.	ORG3
	*Political structure*	In my experience, *Country*’s national policy for NTD control supports community-wide MDA.	ORG4
		I have observed that *Country’s* National NTD Master Plan provides sufficient guidance for delivering community-wide MDA programmes, such as lymphatic filariasis (LF).	ORG5
		I have observed that deworming medicines are acquired centrally and re-distributed to local levels without too much difficulty.	ORG6

## Abbreviations

cRCT: Cluster-randomized control trial
CDD: Community drug distributors
cMDA: Community-wide mass drug administration
IQR: Interquartile range
LMIC: Low- and middle-income country
MDA: Mass drug administration
ORIC: Organizational readiness for implementing change
STH: Soil-transmitted helminths
WHO: World Health Organization
